# Deep metaproteomic mapping of gingival crevicular fluid reveals distinct microbial community at prepubertal and circumpubertal stages

**DOI:** 10.1186/s12903-025-07348-6

**Published:** 2025-11-22

**Authors:** Xue Yang, Rijing Liao, Yan Cai, Jun Wang

**Affiliations:** 1 Department of Pediatric Dentistry, Shanghai Ninth People’s Hospital, Shanghai Jiao Tong University School of Medicine, Shanghai, 200433 P. R. China; 2 Shanghai Institute of Precision Medicine, Shanghai Ninth People’s Hospital, Shanghai Jiao Tong University School of Medicine, Shanghai, 200125 P. R. China; 3 College of Stomatology, Shanghai Jiao Tong University, Shanghai, 200025 P. R. China; 4 National Center for Stomatology; National Clinical Research Center for Oral Diseases; Shanghai Key Laboratory of Stomatology; Shanghai Research Institute of Stomatology, Shanghai, 200011 P. R. China

**Keywords:** Gingival crevicular fluid, Metaproteome, Prepubertal stage, Circumpubertal stage, Oral health

## Abstract

**Background:**

Orthodontic diagnosis and treatment planning are closely associated with the pubertal growth spurt. Previously, we developed a simplified MS-based protocol for deep quantitative analysis of human gingival crevicular fluid (GCF) proteome for skeletal maturity indicators. The purpose of this study is to perform an in-depth and comparative analysis of the GCF metaproteome at prepubertal and circumpubertal stages to aid oral health and skeletal maturity evaluation.

**Methods:**

Based on our previously obtained and published LC-MS data, the GCF metaproteome of 45 children (24 subjects from prepubertal group and 21 subjects from circumpubertal group) were analyzed by searching against the Human Oral Microbiome Database using FragPipe software. Differentially expressed bacterial proteins between two groups were analyzed using Wilcoxon rank sum test. Differentially abundant taxa between two groups were evaluated using linear discriminant effect size (LEfSe) analysis.

**Results:**

A total of 192 genera were identified in GCF. *Neisseria* (*Neisseriales*), *Comamonadaceae*, *Burkholderiales*, *Proteobacteria* and *Betaproteobacteria* were the most abundant bacterial taxa at prepubertal stage. *Firmicutes*, *Saccharibacteria_TM7_[G-1]* (*Saccharibacteria TM7*), B*acterium_HMT349*, *Bacilli* and *Alphaproteobacteria* were the most abundant bacterial taxa of GCF microbiota at circumpubertal stage. Compared to that in the prepubertal group, enrichment of *Firmicutes* (*Bacillus*) was observed in the circumpubertal group.

**Conclusions:**

Based on our developed and already published single-pot, solid-phase enhanced sample-preparation (SP3)-based liquid chromatography (LC) - high-field asymmetric waveform ion mobility spectrometry (FAIMS)-MS protocol for deep quantitative analysis of human GCF metaproteome, we were able to generate the largest dataset of the human GCF metaproteome (14376 bacterial proteins) to date and revealed distinct microbial community at prepubertal and circumpubertal stages. The proposed protocol and findings will be useful to aid oral health and skeletal maturity evaluation for orthodontic diagnosis and treatment planning.

**Supplementary Information:**

The online version contains supplementary material available at 10.1186/s12903-025-07348-6.

## Introduction

The oral cavity is one of the five major parts of the human body (gut, mouth, skin, nasal cavity, and genitourinary tract) that the Human Microbiome Program focuses on [1]. The oral microbial community is relatively stable, with less variation between individuals than skin or gastrointestinal microbiomes [2]. The ecological imbalance of the oral microbiome can not only cause a variety of oral diseases, such as caries, pulp apical disease, periodontal disease, etc., but also be closely related to systemic diseases such as tumors, diabetes, rheumatoid arthritis, cardiovascular diseases, and preterm birth, which has a significant impact on human health [3, 4].

For orthodontic diagnosis and treatment planning, the pubertal growth spurt is closely linked to a rapid increase in mandibular length and offers a wide range of therapeutic modifiability [5]. Six maturation stages can be identified by analyzing the morphology of the second to fourth cervical vertebrae visualized in the lateral cephalogram, including cervical stage (CS) 1 and CS 2 (prepubertal), CS 3 and CS 4 (circumpubertal), and CS 5 and CS 6 (postpubertal) [6]. If the orthodontic treatment is in harmony with the skeletal maturation status of children, a desired result could be achieved with minimal risk of adverse effects. As oral microbiota plays vital roles in the developmental stages of children, the understanding of oral microbiome diversity during the pubertal growth spurt is of great importance for oral and orthodontic health [7].

As a part of the oral fluid, gingival crevicular fluid (GCF), a serum transudate found in the gingival sulcus, plays a special role in maintaining the structure of the junctional epithelium and defending against bacterial infection [8]. It is a mixture of ingredients derived from serum, host inflammatory cells, periodontal structural cells, and oral bacteria [9, 10]. It provides a vital environment for oral microorganisms. GCF may reflect the extent of the host response to a microbial challenge, so the determination of GCF components may provide information for the diagnosis of disease [11]. GCF has been extensively studied for the screening of diagnostic biomarkers for periodontal disease [12–14].

The study of GCF metaproteome is still in its infancy. Recently, from pool GCF samples of 10 young adults (20–30 years), Xiao et al.. identified a healthy GCF microbiome which was dominated by 3082 proteins and 69 genera [15]. Furthermore, they characterized the functional activity of the GCF host proteins and microbiota of periodontitis and identified new host-microbiome signatures of periodontitis [16]. However, current GCF metaproteome research still faces two major limitations. First, sample processing of the GCF metaproteome is usually laborious and often results in sample loss. Therefore, the analytical depth of GCF metaproteome is still relatively limited. Second, the study population of GCF metaproteome was mainly adults. To our knowledge, there is no study on profiling the GCF metaproteome of children during the pubertal growth spurt.

Previously, we developed a simple single-pot, solid-phase enhanced sample-preparation (SP3)-based liquid chromatography (LC) - high-field asymmetric waveform ion mobility spectrometry (FAIMS)-MS (SP3-based LC-FAIMS-MS) protocol for in-depth analysis of human GCF proteome and discovered novel skeletal maturity indicators between prepubertal and circumpubertal stages [17]. The use of SP3 combined with FAIMS simplified GCF sample handling with minimal sample loss, thereby improving analytical coverage of the GCF proteome. In this study, we aimed to further characterize the human GCF metaproteome patterns of children at different developmental stages. Gingival crevicular fluid samples were collected from periodontally healthy subjects of prepubertal and circumpubertal developmental stages, and the difference of the metaproteome between the two groups was investigated. We hope that our study can help to explore the effects of natural changes in the oral ecosystem and assess the baseline microbial profile of children at different developmental stages.

## Experimental section

### Materials and chemicals

Urea, thiolurea, Tris (2-carboxyethyl) phosphine (TCEP), iodoacetamide (IAM), anhydrous ethanol (EtOH), ammonium bicarbonate (ABC), trifluoroacetic acid (TFA) and formic acid (FA) were purchased from Sigma-Aldrich. 3-[(3-Cholamidopropyl)dimethylammonio]−1-propanesulfonate (CHAPS) was purchased from Tokyo Chemical Industry Co., Ltd. Trypsin (sequencing-grade) was purchased from Promega. Acetonitrile (ACN, LC/MS grade) was obtained from Fisher Chemical. HEPES buffer (1 M, pH 7.2) was obtained from Thermo Fisher Scientific. Sera-Mag™ SpeedBead Carboxylate-Modified [E7] Magnetic Particles (50 mg/mL, product no. 45152105050250, lot 17163797) and Sera-Mag™ SpeedBead Carboxylate-Modified [E3] Magnetic Particles (50 mg/mL, product no. 65152105050250, lot 17119044) were purchased from Cytiva. Absorbent paper points were purchased from Meta Biomed Co., Ltd. Ultra-pure water was obtained from a Milli-Q Water System (Millipore).

### Study population

All GCF samples were collected from subjects at Shanghai Ninth People’s Hospital, affiliated with Shanghai Jiao Tong University School of Medicine. All children were free of untreated dental caries, underwent supragingival scaling, and received daily oral hygiene management under parental supervision. Inclusion and exclusion criteria were as follows: (1) children before and during pubertal growth spurt, (2) good oral hygiene and periodontal health, (3) no syndrome or developing mental abnormality of facial structures, (4) no history of orthodontic treatment, trauma or surgery on facial structures, (5) no systemic diseases, (6) had not taken any medication in the past 3 months. Good oral hygiene and periodontal health are defined as follows: during the examination, there is no visible soft debris, no calculus, no untreated caries, no gingival redness or bleeding, no significant alveolar bone resorption on panoramic radiographs, and no clinical evidence of periodontal attachment loss. We used standardized indices to assess the oral hygiene and periodontal conditions of each child.

**Fig. 1:**
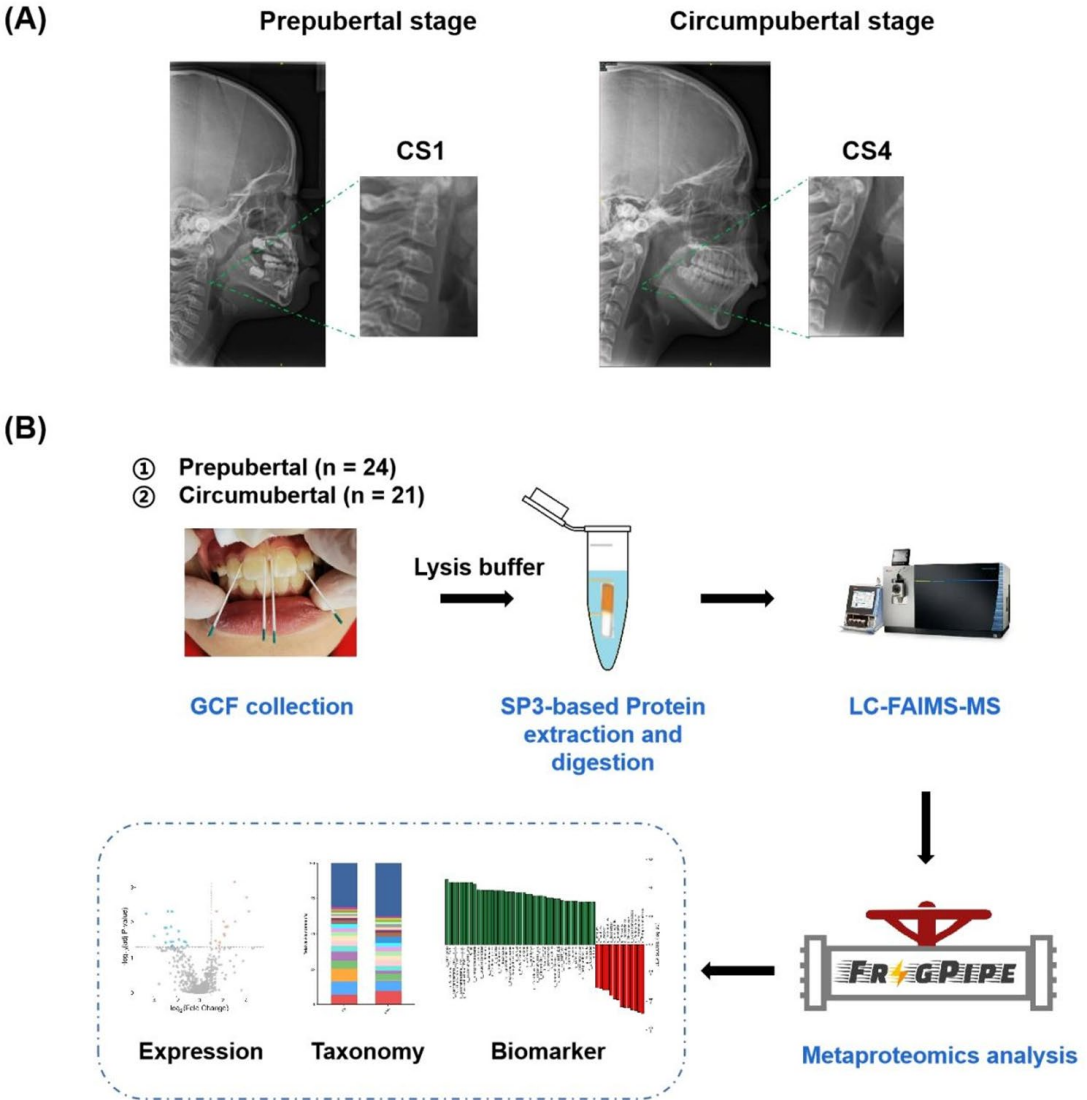
Schematic view of the study. (**A**) Typical lateral cephalograms of participating subjects at the prepubertal (CS1 and CS2) and circumpubertal (CS3 and CS4) stages. (**B**) The workflow of in-depth analysis of GCF metaproteome by SP3-based LC-FAIMS-MS platform. The distinct GCF microbial community at prepubertal and circumpubertal stages will aid oral health evaluation for orthodontic diagnosis and treatment planning

Lateral cephalograms of all subjects were recorded to determine the stage of craniofacial skeletal maturation (Fig. 1A). The cervical vertebral maturation (CVM) method is used to determine the craniofacial skeletal maturational stage of an individual during the growth. Based on the examination of the morphological changes in the second (C2) to fourth (C4) cervical vertebrae, the growth process could be divided into six stages, including cervical stage (CS) 1 and CS 2 (prepubertal), CS 3 and CS 4 (circumpubertal), and CS 5 and CS 6 (postpubertal) [6]. Briefly, when the lower border of C3 shows an indentation, the lower border of C4 is flat, and the shape of C3 and C4 is conical or horizontally rectangular, this is defined as the CS3 stage, which corresponds to the pubertal period. When the lower border of C4 shows an indentation and both C3 and C4 are horizontally rectangular, this is defined as the CS4 stage, which marks the end of the pubertal period or indicates that it has ended within the previous year. Consequently, the CS3 and CS4 stages are collectively referred to as the circumpubertal period. The CS1 and CS2 stages correspond to the prepubertal period, while the CS5 and CS6 stages correspond to the postpubertal period. To minimize observer bias, the same orthodontist (Xue Yang) interpreted the same cephalogram at one-week intervals and performed three readings in total. The patient’s cervical vertebral maturation stage was recorded only when the results were consistent across the three readings.

### GCF collection and sample preparation

Before GCF collection, the sampling area was isolated with cotton rolls and dried with a gentle air stream to prevent contamination from saliva. Absorbent paper points were gently inserted into the gingival crevice or periodontal pocket and left in place for 30 s (Fig. 1B). Mechanical irritation was avoided and paper points visibly contaminated with blood were thrown away. The wetted part of paper points was then placed into an Eppendorf tube and 100 µL of lysis buffer (7 M urea, 2 M thiourea, 5 mM TCEP, 2% CHAPS and 20 mM HEPES) was added. After incubation at room temperature for 10 min, the sample underwent a centrifugation at 12,000 g and the supernatants were subsequently transferred to another Eppendorf tube and stored at −80 °C. On the day of analysis, the concentration of total GCF protein was measured using the Detergent Compatible Bradford Assay Kit (Beyotime Biotechnology) according to the manufacturer’s instructions on a Thermo Scientific Multiskan FC microplate photometer.

To each GCF sample, TCEP was added to a final concentration of 10 mM and the reduction reaction was kept for 10 min at room temperature. IAM was then added to a final concentration of 20 mM and the alkylation was performed for 40 min in the dark at room temperature. Before use, a 1:1 combination of two different types ([E3] and [E7]) of SP3 beads was rinsed twice with water with help of a magnetic rack. For SP3 binding, EtOH was added to a specific final concentration (70% v/v). Subsequently, SP3 beads were added to the GCF samples at a ratio of 20:1 (beads to proteins) and ~ 300 µL of the mixture was incubated at 1000 rpm for 10 min at room temperature. After SP3 binding, the supernatant was removed and the SP3 beads were rinsed three times with 180 µL of 80% EtOH. For SP3 peptide elution and recovery, SP3 beads were resuspended in 100 µL of 25 mM ABC, followed by the addition of trypsin (1:25, enzyme to protein) and incubated overnight at 37 °C at 1000 rpm. After digestion, the supernatant was transferred to a new Eppendorf tube. The recovered peptides were acidified by the addition of TFA to a final concentration of 0.5% and subjected to C18 desalting using homemade StageTips (pre-packed with the Empore C18 membrane). The desalted peptides were lyophilized for further LC-MS analysis.

### Nano LC-MS/MS analysis

All the nano LC-MS/MS analysis was performed on an Easy UPLC system coupled to an Orbitrap Fusion Lumos mass spectrometer (Thermo Fisher Scientific). The lyophilized peptides were resuspended in buffer A (0.1% FA in water). The samples were loaded and then separated on a homemade analytical column (C18, 75 μm×15 cm) at a flow rate of 250 nL/min over a 150-min gradient (buffer B, 0.1%FA in 80%ACN, 0–136 min, 5 to 40% B, 136–144 min, 40–80% B, 144–150 min, 80%B). The column temperature was set at 55 °C. The electrospray voltage was set at 2.1 kV and the temperature of the ion transfer tube was set at 275 °C. The S-lens RF level setting was 40%. For each 3 s cycle of duty, it consisted of one full-MS survey scan (375–1500 m/z) with a resolution of 60 K and then MS/MS scans with a resolution of 15 K. For MS, the AGC was set to 4e^5^ ions, with maximum accumulation times of 50 ms. For MS/MS, HCD was used with 30% normalized collision energy, an isolation window of 1.6 m/z, and the AGC was set to 5e^4^ ions, with a maximum accumulation time of 35 ms. 2–6 charge state was included and dynamic exclusion was 60 s. The FAIMS Pro duo device was placed between the nano electrospray source and the mass spectrometer. All the analysis with FAIMS had a cycle time of 1 s per CV. FAIMS separations were performed with the following settings: inner and outer electrode temperature = 100 °C, FAIMS carrier gas flow = 0 L/min, dispersion voltage = −5000 V, entrance plate voltage = 250 V. The combination of three CVs were − 45/−60/−75 V.

### Data analysis

The MS/MS data were searched against the Human Oral Microbiome Database V3.1 (downloaded from the eHOMD website http://www.ehomd.org, 22,980,317 sequences) and human UniProt database (20,589 sequences) using FragPipe (Version 21.1). The search settings were as follows: enzyme, trypsin, max missed cleavages, 2, digest mode, specific, fixed modification, the carbamidomethylation of Cysteine (57.02 Da), variable modifications, the oxidation of Methionine (15.99 Da) and the acetylation of the protein N-term (42.01 Da), parent mass error tolerance, 10 ppm, fragment mass error tolerance 20 ppm. The false discovery rates (FDRs) of the peptide-spectrum matches (PSMs) and proteins identification were set to less than 1%. The unique peptides were set to no less than 3. The proteins expressed in more than 50% of all samples were included. For the quantitative analysis, label-free quantification (LFQ) was performed based on Top3 unique peptide intensity of each protein.

‘Wu Kong’ platform (https://www.omicsolution.com/wkomics/main/) was used for statistical analysis and bioinformatics analysis of differentially expressed bacterial proteins. The proteins expressed in less than 50% of each group were excluded. After data median normalization, the remaining missing values were imputed with the global minimum value of the dataset. To assess the distinction of metaproteomic data from two groups, sparse partial least square-discriminant analysis (sPLS-DA) was performed using MetaboAnalyst 6.0. For differentially expressed bacterial proteins analysis, the Wilcoxon rank sum test (non-normally distributed data) was used to compare data between prepubertal and circumpubertal groups. Up- or down-regulated bacterial proteins were defined as bacterial proteins differentially expressed in the circumpubertal group compared to the prepubertal group (fold change > 2 or < 0.5, Benjamini-Hochberg adjusted *P* < 0.05). For differentially abundant taxa analysis, the abundance of each taxa was represented as the sum of the LFQ intensities for all bacterial proteins. LEfSe (linear discriminant analysis effect size) was then performed using the OmicStudio tools at https://www.omicstudio.cn/tool. GraphPad Prism 8.0, Figtree 1.4.4 and the online tool (https://www.bioinformatics.com.cn) were used for data visualization.

## Results

### Clinical characteristics of children at two developmental stages

According to inclusion and exclusion criteria, a total of 45 children were included in this study. According to the CVM method, 24 subjects were from the prepubertal group (13 males and 11 females, mean age 8.2 ± 2.2 years) and the other 21 subjects were from the circumpubertal group (9 males and 12 females, mean age 12.4 ± 1.0 years). There were no significant differences in gender, plaque index (PLI), bleeding index (BI), extrinsic black tooth stain (EBS), probing depth (PD), filled teeth (FT) and GCF protein amount between the two groups (*P* > 0.01) (Table 1).

**Table 1:** Clinical features of the study population

Items	Prepubertal (*n* = 24)	Circumpubertal (*n* = 21)	*P* value
Gender (male/female)	13/11	9/12	0.46^a^
Chronological ages (years)	8.2 ± 2.2	12.4 ± 1.0	0.00^b^
CVM stage	CS1(16), CS2(8)	CS3(12), CS4(9)	
Hellman stage			
II A	3	0	
II C	1	0	
III A	14	0	
III B	4	0	
III C	0	11	
IV A	2	10	
FT	2.7 ± 3.1	0.7 ± 0.9	0.02^b^
0	9	11	
1–4	10	10	
5–8	2	0	
9–20	3	0	
PLI			0.97^b^
PLI = 0	15	13	
PLI = 1	9	8	
BI			0.89^a^
BI = 0	19	17	
BI = 1	5	4	
PD			0.54^a^
PD = 1	7	8	
PD = 2	17	13	
PAL	0	0	n/a
EBS			0.56^a^
Negative (0)	21	17	
Positive (1)	3	4	
GCF protein amount (µg/strip)	6.3 ± 1.9	8.2 ± 3.5	0.04^b^

BI, bleeding index; EBS, extrinsic black tooth stain; PLI, plaque index; PD, probing depth; PAL, periodontal attachment loss; FT, filled teeth

a T-test

b Wilcoxon rank sum test

### Comparative analysis of GCF metaproteome between prepubertal and circumpubertal groups

**Fig. 2:**
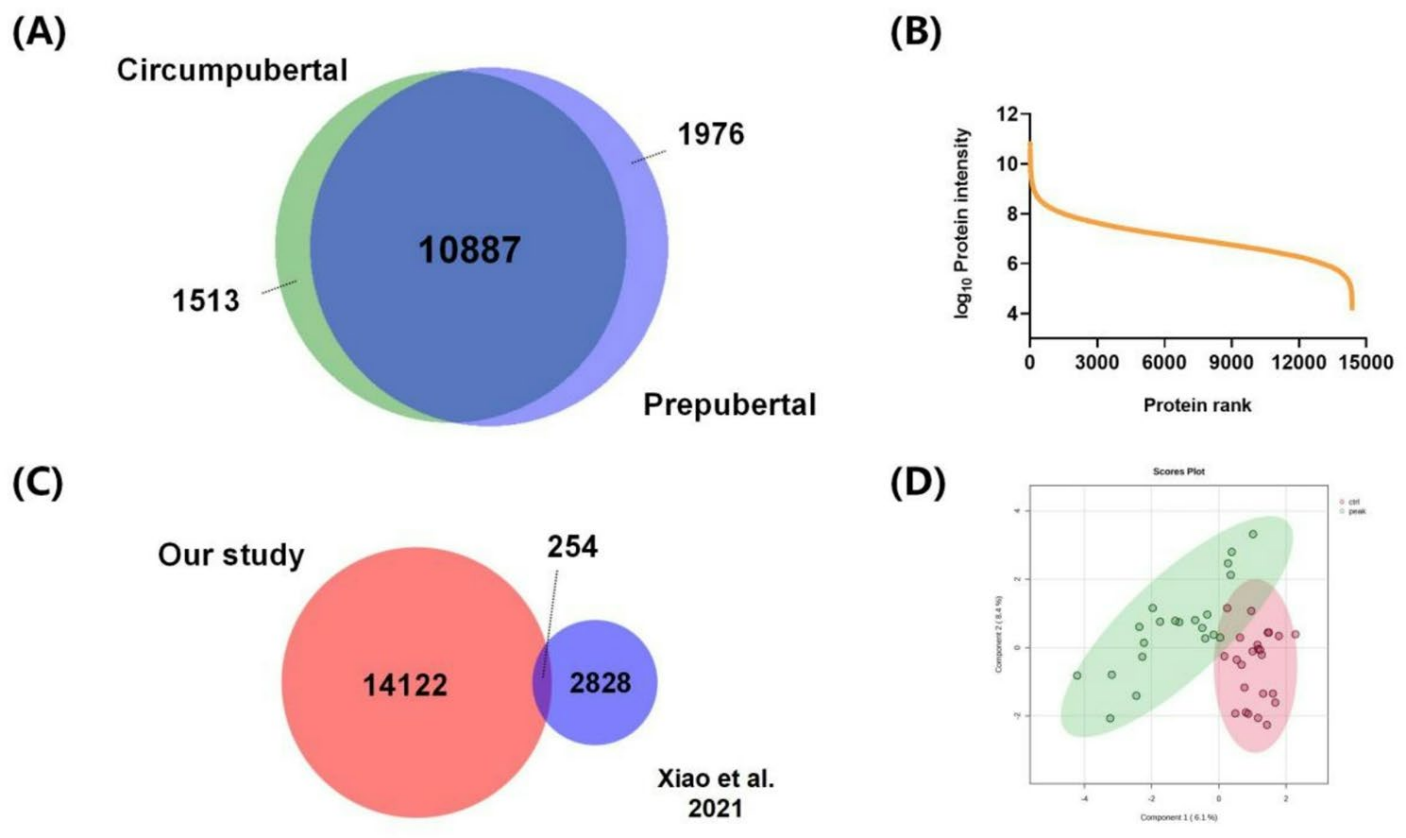
Deep profiling of GCF metaproteome from prepubertal and circumpubertal groups through SP3-based LC-FAIMS-MS platform. (**A**) Venn diagram of the GCF bacterial proteins identified in prepubertal and circumpubertal groups. (**B**) Dynamic range of protein abundance of the GCF metaproteome at the prepubertal and circumpubertal stages. Protein abundance was calculated by averaging the total intensity of a given protein across all GCF samples (*n* = 45). **(C**) Comparison of the depth of the GCF metaproteome identified in our study and the recent paper (Xiao et al., Proteomics 2021, 21, 2000321). (**D**) Scores plot for sparse partial least square-discriminant analysis (sPLS-DA) of the GCF metaproteome between prepubertal and circumpubertal groups. Prepubertal group (ctrl) was shown in the red circle and circumpubertal group (peak) was shown in the green circle

A total of 14,376 bacterial proteins was identified in all GCF samples, of which 12,863 bacterial proteins were identified in the prepubertal group (*n* = 24) and 12,400 bacterial proteins were identified in the circumpubertal group (*n* = 21) (Fig. 2A). The detailed information of the identified bacterial proteins are listed in Table S1. In this GCF metaproteome, their abundance spanned a dynamic range of eight orders of magnitude (Fig. 2B). 60 kDa chaperonin 1 were found to be highly abundant, which was in good agreement with previous reports on the human GCF metaproteome profile [15].

**Fig. 3:**
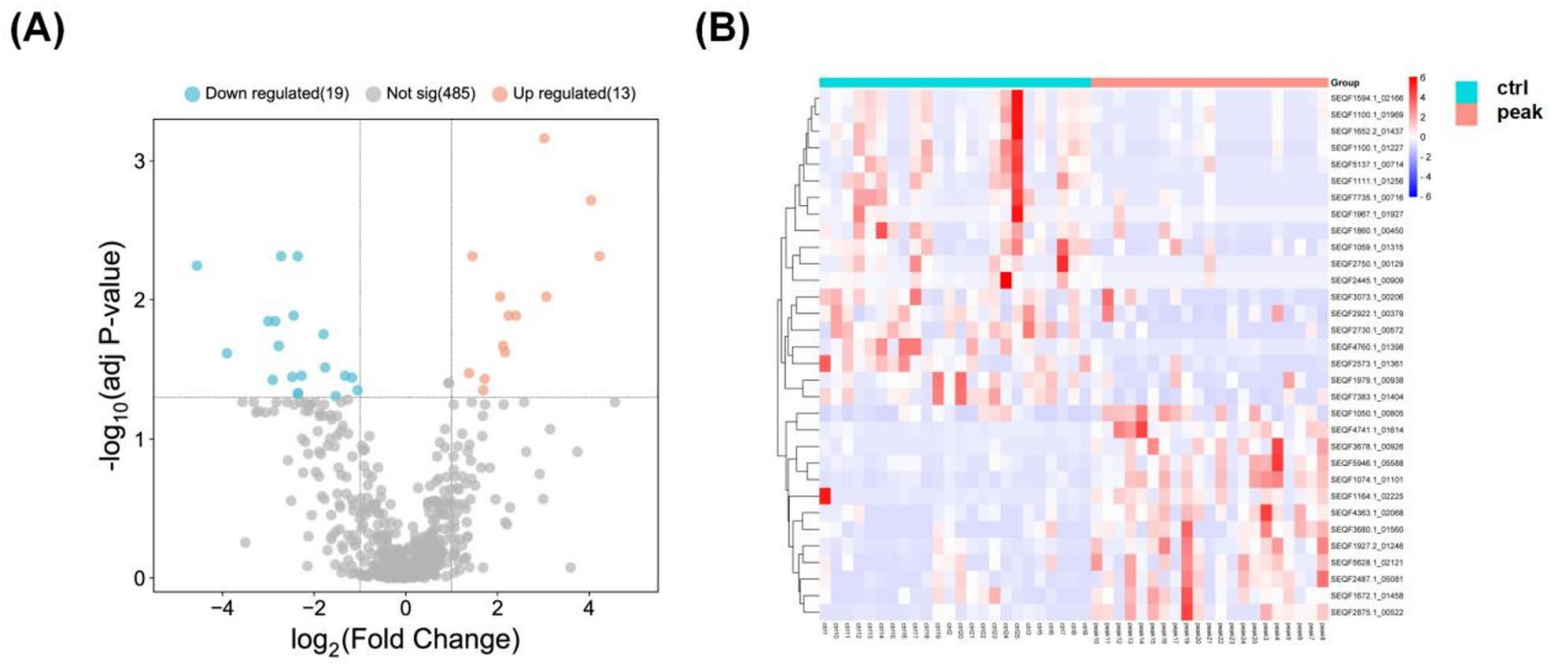
Label-free quantification of the GCF metaproteome between prepubertal and circumpubertal groups. (**A**) The volcano plot illustrates differentially expressed proteins between prepubertal and circumpubertal groups (Fold change > 2, adjusted *P* value < 0.05). Vertical dashed lines denoted a linear fold change of 2 in either direction (prepubertal group/circumpubertal group or circumpubertal group/prepubertal group). The horizontal dashed line indicates a cutoff of 0.05 for the adjusted *P* value. (**B**) The hierarchical clustering heatmap illustrates 32 differentially expressed proteins between prepubertal and circumpubertal cohorts. Ctrl indicates prepubertal cohort and peak indicates circumpubertal cohort

The abundance of bacterial protein in prepubertal and circumpubertal groups was compared by label-free quantification. After data filtering and imputation, 517 bacterial proteins were retained for subsequent statistical analysis. As shown in Fig. 2D, sPLS-DA illustrated a clear distinction between the GCF metaproteome of prepubertal and circumpubertal samples. Volcano plots revealed that bacterial proteins were differentially expressed between prepubertal and circumpubertal groups, of which 13 bacterial proteins were up-regulated and 19 bacterial proteins were down-regulated in the circumpubertal group (fold change > 2 or < 0.5, adjusted *P* < 0.05) (Fig. 3A). The detailed information of these differentially expressed GCF bacterial proteins are listed in Table S2. The expression pattern of these differentially expressed bacterial proteins between prepubertal and circumpubertal groups was also clearly showed in the hierarchical clustering heatmap (Fig. 3B). GO enrichment analysis and KEGG pathway analysis were performed on 32 differentially expressed bacterial proteins between prepubertal and circumpubertal groups. However, when adjusted *P* < 0.05 was selected, no enriched pathway was observed (data not shown).

A total of 6128 human-derived proteins was identified. This number is comparable to the 5407 human proteins reported in our previously published paper [17]. These data will not be shown or discussed in this study.

### Taxonomy overview and microbiota changes between prepubertal and circumpubertal groups

**Fig. 4:**
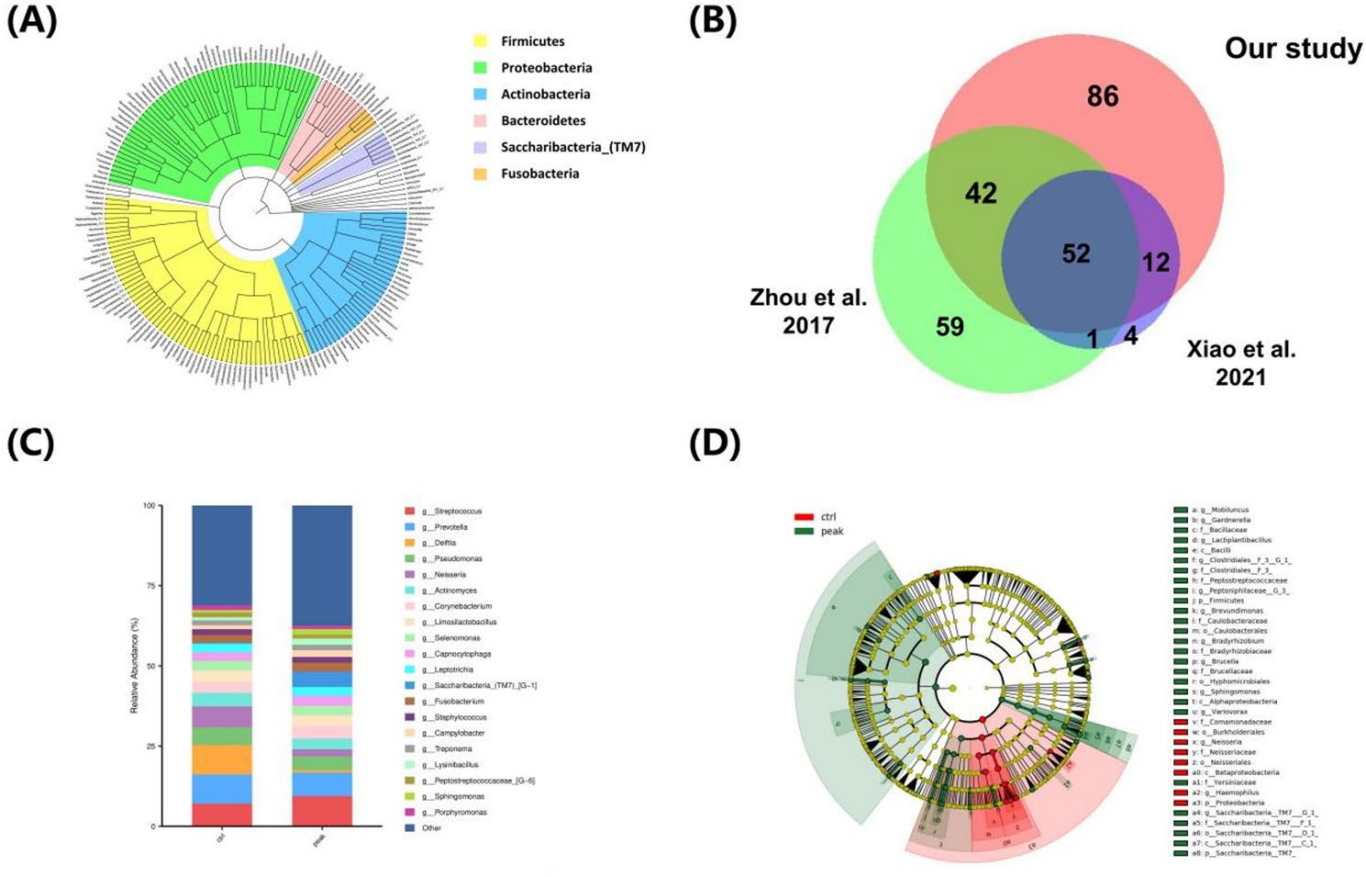
Taxonomy overview and microbiota changes between prepubertal and circumpubertal groups. (**A**) Taxonomy tree of 192 bacterial genera. (**B**) Comparison of the GCF bacterial genera identified in our study and recent papers (Zhou et al., Front. Microbiol. 2017, 8, 2144 and Xiao et al., Proteomics 2021, 21, 2000321). (**C**) Relative quantification of the GCF bacteria (TOP 20 genera) between prepubertal and circumpubertal groups. (**D**) LEfSe analysis of the identified bacteria, significantly more abundant in prepubertal group (ctrl, red) and circumpubertal group (peak, green). The circular cladogram presents the predominant taxa of GCF microbiome in each group

At the phylum level, GCF microbiota mainly included *Firmicutes*,* Proteobacteria*,* Actinobacteria*,* Bacteroidetes*,* Saccharibacteria_(TM7)* and *Fusobacteria* (Fig. 4A), which was in agreement with the previous report on human oral microbiome [18] At the genus level, a total of 192 genera were identified, covering most of recently reported GCF genera and 86 genera were unique in this study (Fig. 4B) [15, 19]. This result indicated that our GCF microbiome data could be a solid complement to the current GCF metaproteome. As shown in Fig. 4C, for the most abundant genera, *Delftia*, *Prevotella*, *Streptococcus*, *Neisseria* and *Pseudomonas* were the top five ones in prepubertal group, while in the circumpubertal group, they were *Streptococcus*, *Prevotella*, *Saccharibacteria_(TM7)_[G-1]*, *Pseudomonas*, *Corynebacterium*. The above results showed that the two groups had different genus characteristics. To compare the difference of the bacteria identified in the prepubertal and circumpubertal group, LEfSe analysis was performed. *Neisseria* (*Neisseriales*), *Comamonadaceae*, *Burkholderiales*, *Proteobacteria* and *Betaproteobacteria* (LDA scores (log10) >4) were dominated in the prepubertal group, whereas *Firmicutes*, *Saccharibacteria_TM7_[G-1]* (*Saccharibacteria TM7*), B*acterium_HMT349*, *Bacilli* and *Alphaproteobacteria*, (LDA scores (log10) >4) were the most abundant bacterial taxa of GCF microbiota in the circumpubertal group (Fig. 4D and Fig. S3).

## Discussion

Previously, we developed a simple SP3-based LC-FAIMS-MS protocol for in-depth analysis of human GCF proteome and discovered novel skeletal maturity indicators between prepubertal and circumpubertal stages [17]. In this study, based on this previous work, we aimed to further perform a comparative analysis of GCF metaproteome between prepubertal and circumpubertal subjects to aid oral health and skeletal maturity evaluation.

Totally, we identified 14,376 bacterial proteins in all GCF samples, of which 12,863 bacterial proteins were identified in the prepubertal group and 12,400 bacterial proteins were identified in the circumpubertal group. To our knowledge, this performance is the most comprehensive human oral microbiome profiling and we generated the largest human GCF metaproteome dataset to date (Table S3). Notably, only an overlap of 254 bacterial proteins (1.5%) was observed between our dataset and a recently reported GCF metaproteome (Fig. 2C). Additionally, the number of identified bacterial proteins in each sample varied largely and the correlation of the abundance of GCF metaproteome in all samples was relatively low (Fig. S1 and S2). The above results indicated a great diversity of the human oral microbiome.

With the growth of chronological age and tooth development, the species and quantity of oral microbiota are gradually increasing [20]. For example, Crielaard et al. demonstrated that salivary microbiota of children aged 3 to 18 years is still in the process of maturation [21]. In this work, we provided an effective GCF data foundation to assess the baseline microbial profile of children during the pubertal growth spurt. The distinct microbial community in GCF would be a good complement to a better understanding of oral health status. Additionally, in terms of skeletal maturity evaluation, previous studies have demonstrated that *Firmicutes* (*e.g. Lactobacillus*) in the gut produce short-chain fatty acids, which improve intestinal calcium absorption and inhibit inflammation, thereby indirectly supporting bone mineralisation and bone density [22, 23]. Our results shows the enrichment of *Firmicutes* (*bacillus*) in the circumpubertal group compared to that in the prepubertal group, indicating its potential to be a candidate indicator of skeletal maturity for orthodontic diagnosis and treatment planning.

One limitation of this study is its relatively small sample size. Given the high diversity of GCF microbiota and subject variability in the composition of GCF, if the sample size increases, rarer species might be discovered, and the differential bacterial proteins would be validated and evaluated. Therefore, in the future, with the development of high-throughput metaproteomics techniques, more time-course validation studies with larger, more diverse cohorts in multicenter are needed to be conducted.

## Conclusion

In summary, based on our previously developed SP3-based LC-FAIMS-MS protocol, we performed a deep quantitative analysis of human GCF metaproteome. We generated the largest dataset of the human GCF metaproteome to date and revealed distinct microbial community at prepubertal and circumpubertal stages. The proposed protocol and findings will be useful to aid oral health and skeletal maturity evaluation for orthodontic diagnosis and treatment planning. However, due to the limited number of subjects involved, future large-scale clinical studies are needed to further validate.

## Supplementary Information

Supplementary Material 1.

Supplementary Material 2.

Supplementary Material 3.

## Data Availability

The datasets used and/or analysed during the current study are available from the corresponding author on reasonable request.
