# Supplementary material for: Deep metaproteomic mapping of gingival crevicular fluid reveals distinct microbial community at prepubertal and circumpubertal stages
Source: BMC Oral Health. 2025 Nov 22;25:1974. doi: 10.1186/s12903-025-07348-6 (PMC12751196; doi:10.1186/s12903-025-07348-6)
Supplement: Supplementary file 1 — Supplementary Material 1. [file 12903_2025_7348_MOESM1_ESM.docx]

**Supplementary material**

**Deep metaproteomic mapping of gingival crevicular fluid reveals distinct microbial community at prepubertal and circumpubertal stages**

Xue Yang^1,3,4^, Rijing Liao^2,^*, Yan Cai^2,^*, Jun Wang^1,3,4,^*

^1^ Department of Pediatric Dentistry, Shanghai Ninth People’s Hospital, Shanghai Jiao Tong University School of Medicine, Shanghai, 200433, P. R. China

^2^ Shanghai Institute of Precision Medicine, Shanghai Ninth People’s Hospital, Shanghai Jiao Tong University School of Medicine, Shanghai, 200125, P. R. China

^3^ College of Stomatology, Shanghai Jiao Tong University, Shanghai, 200025, P. R. China

^4^ National Center for Stomatology; National Clinical Research Center for Oral Diseases; Shanghai Key Laboratory of Stomatology, Shanghai, 200011, P. R. China

*Corresponding author:

Rijing Liao, E-mail: [rjliao@shsmu.edu.cn](mailto:rjliao@shsmu.edu.cn;);

Yan Cai, E-mail: [caiyan0723@shsmu.edu.cn;](mailto:caiyan0723@shsmu.edu.cn;)

Jun Wang, E-mail: wangjun202@126.com.

**Figure S1.** The number of identified GCF bacterial proteins from prepubertal (n=24) and cicumpubertal (n=21) samples by SP3-based FAIMS-MS platform.


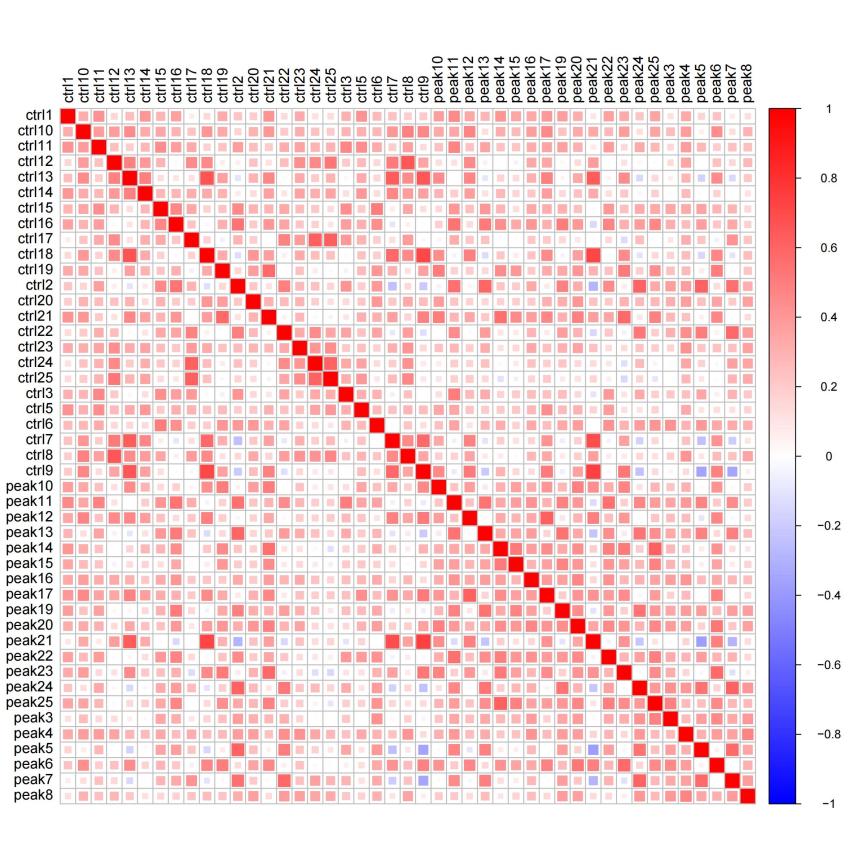


**Figure S2.** Correlation analysis of the abundance of GCF metaproteome in all samples (n=45).


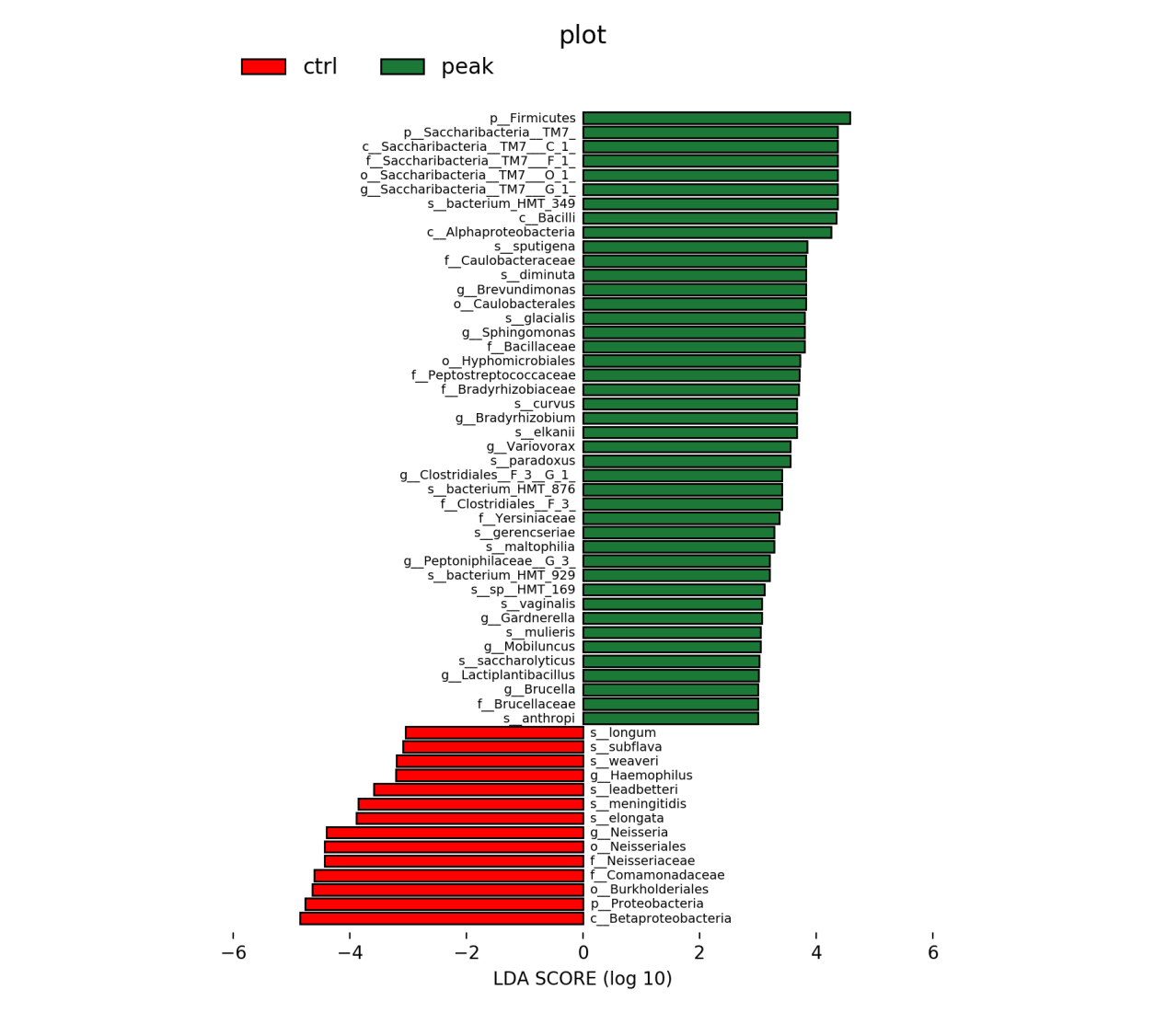


**Figure S3.** LEfSe analysis indicating the difference of identified bacteria between prepubertal group (ctrl, red) and circumpubertal group (peak, green). The threshold on the linear discriminant analysis (LDA) score for discriminative features was set at 3.0.

**Table S1.** The detailed information of the identified GCF bacterial proteins in prepubertal and cicumpubertal groups. (See attached Excel Table S1)

**Table S2.** The detailed information of the differentially expressed GCF bacterial proteins between prepubertal and cicumpubertal groups. (See attached Excel Table S2)

**Table S3.** Comparison of the performance of oral microbiome analysis between our study and recently published reports.

| No. | Sample | MS method | The number of bacterial proteins (genus) | Reference |
| --- | --- | --- | --- | --- |
| 1 | GCF  (n=10) | Triple TOF 5600  DDA | 3082 (69) | Proteomics 2021, 21, e2000321. |
| 2 | GCF  (n=16) | Orbitrap  Fusion Lumos  DIA | 752 (51) | J. Proteome Res. 2023, 22, 2411-2420. |
| 3 | Saliva  (n=34) | Orbitrap   1. Exactive Plus   DDA | 3,647 (249)* | Research (Wash D C) 2022, 2022, 9781578. |
| 4 | Tongue coating  (n=345) | Orbitrap  Q Exactive HF-X  DIA | 13,780 (102) | Microbiome 2024, 12, 6. |
| 5 | GCF  (n=45) | Orbitrap  Fusion Lumos  DDA | 14,376 (192) | Our work |

* 249 indicates the number at specie level. The number at genus level is not shown.
